# Views of rare disease participants in a UK whole-genome sequencing study towards secondary findings: a qualitative study

**DOI:** 10.1038/s41431-018-0106-6

**Published:** 2018-02-13

**Authors:** Michael P Mackley, Edward Blair, Michael Parker, Jenny C Taylor, Hugh Watkins, Elizabeth Ormondroyd

**Affiliations:** 1 Division of Cardiovascular Medicine, Radcliffe Department of Medicine, University of Oxford, Oxford, UK; 2 Department of Clinical Genetics, Oxford University Hospitals NHS Foundations Trust, Oxford, UK; 3 Ethox Centre, Nuffield Department of Population Health, University of Oxford, Oxford, UK; 4 Wellcome Trust Centre for Human Genetics, University of Oxford, Oxford, UK; 5 National Institute for Health Research (NIHR) Comprehensive Biomedical Research Centre, Oxford, UK

## Abstract

With large-scale genome sequencing initiatives underway, vast amounts of genomic data are being generated. Results—including secondary findings (SF)—are being returned, although policies around generation and management remain inconsistent. In order to inform relevant policy, it is essential that the views of stakeholders be considered—including participants who have made decisions about SF since the wider debate began. We conducted semi-structured interviews with sixteen rare disease patients and parents enroled in genome sequencing to explore views towards SF. Informed by extensive contact with the healthcare system, interviewees demonstrated high levels of understanding of genetic testing and held pragmatic views: many are content not knowing SF. Interviewees expressed trust in the system and healthcare providers, as well as an appreciation of limited resources; acknowledging existing disease burden, many preferred to focus on their primary condition. Many demonstrated an expectation for recontact and assumed the possibility of later access to initially declined SF. In the absence of such an infrastructure, it is important that responsibilities for recontact are delineated, expectations are addressed, and the long-term impact of decisions is made clear during consent. In addition, some interviewees demonstrated fluid views towards SF, and suggestions were made that perceptions may be influenced by family history. Further research into the changing desirability of SF and behavioural impact of disclosure are needed, and the development and introduction of mechanisms to respond to changes in patient views should be considered.

## Introduction

Whole-genome and whole-exome sequencing (collectively, ‘genome sequencing’; GS) is expected to transform the investigation of rare disease and cancer [1]. In the UK, GS is being delivered by the National Health Service (NHS) in routine clinical practice through the 100,000 Genomes Project [2]. However, the question of whether to test for ‘secondary findings’ (SF)—variants associated with health conditions other than the condition under investigation—remains unresolved [3, 4]. SF can arise either incidentally during analysis performed to identify the genetic cause of the indication for sequencing, or during an intentional screen of specific additional genes. While of potential benefit, these findings present numerous challenges for healthcare systems, patients and practitioners [5].

In 2013, the American College for Medical Genetics and Genomics (ACMG) published guidelines that recommended screening of a list of genes for variants of known (or expected) pathogenicity in all individuals (including healthy relatives) undergoing clinical GS [6]. Recently updated [7], this list includes genes in which variants could imply risk of potentially life-threatening yet asymptomatic disease, where intervention is available. Other organisations agreed that SF of potential clinical significance should be reported, while encouraging their minimisation by restricting analysis to genes implicated in the patient’s primary condition [8, 9].

GS programmes have differed in their handling of SF: some offer a wider range [10], while others offer fewer or none [11]. The 100,000 Genomes Project offers optional return of actionable SF in a very limited gene list, as well as carrier status for some recessive and X-linked conditions. Lemke et al. [12] argue that engaging stakeholders in the development of such genomic policies can better align practices with societal needs and expectations, increase quality of the developed policies and facilitate guideline uptake. Previous studies showed greater caution towards SF among some patient groups compared to non-patient groups, largely informed by experience with their primary condition and of the healthcare system [13–16]. These studies, however, are largely hypothetical—they do not capture the experience of participants embedded in GS projects, making actual decisions about results [5]. The implementation of specific policies towards SF allows participant perspectives to be explored, with reference to informed consent and actual decisions made. Recently, survey studies have begun to capture such individuals [17], but in-depth qualitative studies are lacking.

We report on a qualitative study aimed at providing in-depth data on understanding, views and experiences of patient and parents enroled in GS studies towards SF. All participants have made actual decisions about SF, affording exploration of the factors underlying their choices. We believe that this is timely as early results from the 100,000 Genomes Project are being returned to participants, including SF, where requested.

## Materials and methods

### Setting

In Oxford, several large-scale GS initiatives preceded the 100,000 Genomes Project [18, 19], recruiting patients and family members with rare diseases through diverse medical specialities. Many patients—affected adults or children, or healthy relatives where appropriate—were recruited using ‘Molecular Genetic and Analysis and Clinical Studies of Individuals and Families at Risk of Genetic Disease’ (MGAC), a protocol that offers adult participants options with respect to SF: medically actionable findings discovered incidentally during analysis, and screening of a gene list for ‘additional findings’ based on ACMG recommendations [6]. Fully informed consent was obtained in the context of genetic counselling by a medical specialist or genetic counsellor involved in care of the patient or family; sessions took between 30 and 90 min. Interviews took place between 11 and 24 months after enrolment in the main study. In the intervening period, some participants had received primary findings.

### Study participants

At the time of enrolment, participants were offered the option of participating in a social sciences sub-study. Adult GS participants (age 18 or older, including parents) who consented to be contacted were eligible to participate in an interview.

### Recruitment and data collection

**Fig. 1:**
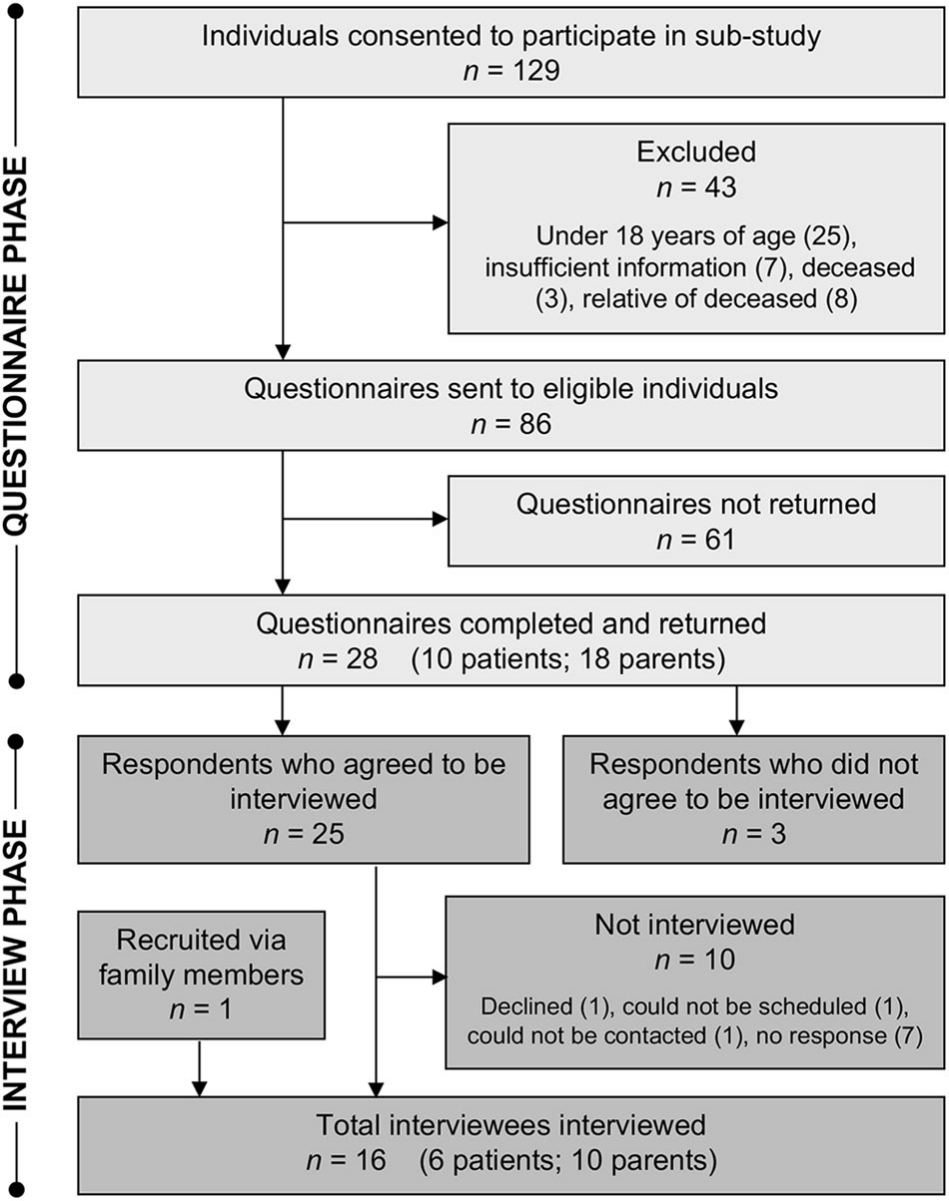
Recruitment pipeline for questionnaire and interviews

All eligible participants (*n* = 86) were approached via a letter explaining the purpose of the sub-study with a questionnaire to elicit biographical data and opinions on the wider GS study (Fig. 1; questionnaire data not reported). Participants willing to be interviewed were contacted by email or telephone. Using a semi-structured interview guide (Supplementary Information), MPM (trained in qualitative research, unknown to interviewees; blind to participant medical history and consent decisions at time of interview) conducted interviews between March and November 2016, lasting 25–60 min, at a location of interviewee’s choosing. All participants had capacity to provide consent. Data analysis was carried out in parallel with data collection and the interview guide modified as interviews progressed. Written informed consent was obtained under the MGAC study protocol, approved by the West Midlands Research Ethics Committee, reference 13/WM/0466.

### Data analysis

Interviews were audio-recorded, transcribed verbatim, anonymised and checked for accuracy. A thematic analysis was then undertaken [20]. This was inductive and experiential, aiming to provide a rich description of the data set, prioritising participant interpretation, views, and perspectives. Initially, the first and last author independently coded three transcripts in a non-restricted matter and discussed. The remaining transcripts were then coded by the MPM using NVivo 11 (QSR International, Melbourne). The coding structure was revised iteratively throughout the analysis. On the basis of the grouping of codes, MPM and EO arrived at higher-level themes and subthemes. Transcripts were then re-read to verify concordance with the data and to select illustrative quotations (presented below with anonymised codes; pronouns random to protect identity). Where possible, reporting follows the COREQ checklist for qualitative research [21].

## Results

28 questionnaires were returned (33%); 25 respondents agreed to be interviewed. Of these, 15 interviews were arranged (60%). A further interviewee was recruited via a family member. Thus, a total of 16 individuals were interviewed (Table 1); data saturation was reached within the limitations of the sample. Interviews were conducted in the interviewee’s home (*n* = 11), by telephone (*n* = 4), or the interviewer’s workplace (*n* = 1). As some interviewees are related, a total of 12 families are represented. Through the thematic analysis, four themes were identified.

**Table 1:** Demographics of interviewees (*n* = 16)

	*n*	%
*Gender*		
Male	7	44
Female	9	56
*Affected*		
Yes	5	31
No (parent)	11	69
*Ethnicity*		
White British	16	100
*Education*		
to 16	2	13
to 18	2	13
Degree	6	38
Post degree	6	38
*Age*		
Under 20	1	6
20–50	9	56
50–70	5	31
Over 70	1	6
*Disease area* ^a^		
Cardiology	5	31
Neurology	7	44
Clinical Genetics	4	25
*Primary result*		
Positive	4	25
Negative	3	19
No result yet	9	56

^a^Cardiology cases include cardiomyopathies and congenital heart disease; neurology cases include epilepsies, hereditary spastic paraplegia, and chronic inflammatory demyelinating polyneuropathy (CIDP); clinical genetics cases include arthrogryposis and osteogenesis imperfecta.

### Motivations and understanding: informed by experience

#### Primary condition and the diagnostic odyssey

Interviewees described parallel experiences with the diagnostic process: most recounted multiple genetic and clinical tests they, or their child, had undergone in a search for a diagnosis prior to enroling in GS. This was described as ‘very difficult’ (R14) and ‘quite a long process’ (R15). Most described similar periods of uncertainty and unpredictability in the absence of a diagnosis. This exhaustive process ultimately resulted in their being offered GS.

#### Seeking an answer and desire for control

For most interviewees, their primary goal was to find ‘an answer’ (R16). Some felt this would inform treatment, however, many were aware that a genetic explanation would have no such impact: ‘In terms of an actual cure, or a treatment pathway, I didn’t expect that’ (R2). These individuals described other motivations, including: planning for the future; reproductive decision-making; utility for family; or improving their child’s care.

*I understand that even if we do find out a name it still might not have any bearing on treatment or prognosis or anything like that, but […] it’s quite difficult to try and get extra support for a child even if they need it because of the fact they haven’t got a name [for their condition]. (R15)*

For many, the need to find an answer appeared to contribute to their search for control: to understand how the condition might progress; to help to manage the condition to the benefit of the patient, as well as the family; and, to offer options to prevent recurrence.

Four participants had received a primary finding (the genetic cause of the condition for which they participated in sequencing); responses differed: for some, it was ‘a relief’ (R1), while others were fearful that knowledge of causation could ‘[not] be good news’ (R3). When R3 ultimately received a result, he found the experience and remaining uncertainty anxiety-inducing: ‘I just felt I wanted more information […] is this good news or is it bad news?’ (R3).

#### Understanding of GS, and perception of risks

Informed by their experience with rare disease and often extensive contact with genetic services, interviewees in general had a high level of understanding of GS. Some even demonstrated an appreciation for the nuances—and limitations—of genetic information, including variations on complex concepts such as reduced penetrance, variable expressivity, and the role of environment:

*There’s so many other influencing causes that can exacerbate or minimise the effect of that. Some people say that you can have a genetic disposition for things […] but actually there’re so many other factors that influence that actually you still don’t know what’s going to happen. (R13)*

One interviewee reported a lack of understanding, ‘No, I didn’t know an awful lot’ (R12), while some perceived their own appreciation as enhanced compared to others’:

*I think the trouble is, there’s an awful lot of people, to be honest, in this situation, don’t understand, because most people don’t have the understanding […] so consequently, I’m not sure how they make that decision, but that’s a reflection on other people, not on myself. (R8)*

Some interviewees did not perceive any risks associated with GS: ‘Personally, I didn’t have any worries’ (R2). However, well informed about GS, most were also aware of implications. Indicative of the quality of their pre-test counselling, interviewees often raised the potential for additional results without prompting: ‘I guess the risk with that is, obviously, it could throw up other things’ (R10). A few also raised concerns about discrimination/insurance, but felt that ‘any risks were outweighed by the huge potential benefits’ (R5).

### Desirability of secondary findings

#### Actionability, and motivations to receive SF

Many interviewees clearly recalled discussions around SF when consenting to GS, although, not all were clear about their actual decision. Of those who recalled opting to receive SF, some articulated a perceived right to information or highlighted the importance of reciprocity in research:

*If that data is available, and if that data is about you, yes, I just kind of feel you should be able to have it, if you’ve agreed to participate in the study. (R14)*

For most who recalled opting to receive SF, motivations revolved around potential usefulness for themselves and their family members or wider community—interviewees wanted SF for which they felt clear action could be taken. Individual perceptions of utility, however, varied widely—attitudes of this sample, who have made decisions about SF, aligned with previously described variability in personal definitions of ‘actionability’ among other stakeholder groups [5]. Most interviewees who wanted SF were interested in those where action could be taken to manage or control the related condition. Some expressed interest in receiving all findings, regardless of actionability or certainty. These individuals, based on experience, felt they could cope with adverse prognoses and could plan as a result: ‘[W]ith conditions that there aren’t treatments for there are still ways, positive ways of life and making the most of life.’ (R15) Most others were less inclined to receive non-actionable SF—many felt such results would cause unnecessary anxiety. Results of uncertain significance were widely considered unhelpful for similar reasons.

The most commonly desired type of SF was carrier status. Interviewees felt that ability to inform reproductive decisions was particularly useful, questioning the policies of the current study (which does not return such results): ‘I think if I was a carrier for cystic fibrosis I would want to know’ (R3).

#### Focus on primary condition, and motivations against receiving SF

Nearly half of the interviewees recalled opting not to receive SF at consent. Later comparison with consent forms, however, showed that some interviewees who recalled declining, and were able to articulate reasoning, had actually elected to receive SF at the time of enrolment.

As a result of time spent without a diagnosis, interviewees had experienced consequences of decision-making with incomplete information. Hoping to avoid having to manage uncertain findings, some expressed concern about the analysis process and supported limiting return of SF: ‘I think given the situation where we are in terms of our knowledge it’s the safest and the best policy’ (R9). Most who recalled declining SF felt additional results would cause anxiety: ‘We just took the decision that we would rather live our lives in blissful ignorance than be perhaps weighed down by the risks of ‘what if?’ (R5) In discussing motivations for declining, interviewees often raised the health or psychosocial burden of the primary condition:

*More than anything else, I was worried that […] they’d find something genetically abnormal, and that our son was carrying a heavy enough load in terms of anxiety and blame and guilt […] then he would just worry himself to the grave that he had it too. I felt he had enough of a bad hand-out to him. (R9)*

With knowledge of the impact of rare disease, many appreciated the potential impact of an additional diagnosis and preferred to focus on the primary outcome:

*We took the decision that rather than potentially find out a list of other issues that could affect us, that we would rather focus on the one issue we knew was affecting us. (R5)*

#### Context-dependent desirability of SF

In discussing which SF they might want, interviewees acknowledged that, drawing on personal context, the impact on family should be considered, and some felt relatives should be involved in initial decision-making. Others highlighted that at different stages in one’s life—such as before or after reaching reproductive age—SF may be more or less relevant and therefore desirable. Older age variably influenced preferences: one interviewee said, ‘I’m old enough to take it’ (R12), whereas another older interviewee felt that because of his age, SF were no longer of relevance; if he were younger, he would be more inclined to want SF.

Of note, for some interviewees, family history (or a lack thereof) appeared to influence desire to receive SF: ‘[My spouse] would like to know [about SF] […] because his father died of prostate cancer quite young’ (R3). Family history also influenced anticipated response to potential SF—together with a lack of symptoms, some indicated they would struggle to take significant action in its absence:

*I would actually find it very hard to have a mastectomy if I didn’t actually have a problem […] as a preventative thing […] If I’d got two sisters, an aunt who’d all been through it and had it then, yes, I think I might think quite differently. (R10)*

### Communication of secondary findings: who, when, how

#### Disclosure of SF and the need for a plan

Interviewees felt that disclosure should be face-to-face, in a timely manner, from someone familiar. Most importantly, interviewees stressed the importance of specialist input and being advised what to do next. This is consistent with overarching desire for control over their primary condition. In light of experience with rare disease, they would want timely control over subsequent diagnoses to minimise anxieties discussed with respect to the diagnostic odyssey.

#### Family and information sharing

When first discussing sharing SF, some felt they would only tell immediate family. Only after prompting about the shared nature of genetic information did these interviewees say they would share with the wider family. Despite a sound understanding of genetics, this may indicate that some still instinctively consider genetic results to be personal: ‘that’s my information to share’ (R13). Speaking hypothetically, some parents indicated that (despite consenting to receive SF themselves) they would control access and filter information in order to minimise anxiety for their children:

*Knowing [my son] may get a disease, which isn’t treatable, […] I can’t do anything about it, neither can he; so it’s really a waste […] Bowel cancer, I’d tell them. Alzheimer’s, I probably wouldn’t. (R11)*

For another, the potential negative impact on children led them to decline SF altogether.

*You have to have the right mentality to be able to cope with that […] I don’t think our son would have had it. I think I probably could have, but I was being a little bit more altruistic about my son. (R9)*

### Systemic factors: healthcare professionals and the healthcare system

#### Confidence in the NHS

Throughout the interviews, interviewees repeatedly endorsed the NHS, often informed by their own experience. Trust in the healthcare system appeared to inform views on management of SF. Consequently, many trusted healthcare professionals (HCPs) to make decisions around SF. With awareness of the developing science, some interviewees even expressed comfort allowing the specialist responsible for their care to make decisions about SF on their behalf: ‘They know what’s best at the end of the day […] so, I trust them enough to make those decisions for me.’ (R6)

Interviewees who were more inclined to want more SF—regardless of actionability or certainty— had experienced variability in knowledge of some HCPs, and were less willing to give up responsibility in decision-making around SF:

*As a parent of two children with a genetic condition, we know far more about that condition than, actually a lot of doctors who specialise in other things […] I think, ordinarily, you would put your trust in them, and actually that might not be the best thing to do, because they don’t necessarily know everything. (R14)*

#### Limited resources

Many interviewees spontaneously noted resource pressures on the NHS: the high level of interviewee understanding included an appreciation for the challenges of the analysis process: ‘I think there’s got to be a point of stopping, hasn’t there?’ (R15). Aware of the complexities of delivering GS, interviewees questioned the justification of using resources needed to handle SF:

*So is there a risk that there’s a huge amount of money going on treating healthy people who may never get sick? In a time when the NHS is struggling for funding, I wonder whether or not that would be a good way of using money. (R5)*

As a result, many felt that options for SF should be restricted: ‘It’s not a sweet shop; it’s a medical service’ (R15). Consistent with strong desire to find an answer for their rare disease, some felt that limited resources further justified focusing on primary findings.

#### Expectation of recontact and continuity

Many interviewees demonstrated an expectation that they could be recontacted in light of new information. Raising this unprompted, many—even those who declined SF—felt results would be kept on file should they choose to access them: ‘[W]e haven’t asked to be told any [SF]. We’ve got the information there, it is accessible to us should, at a later date, we change our minds’ *(R8)*. There was a sense among some interviewees that research would continue and their data could be re-interrogated for SF in the future. Some, however, appreciated the challenges, highlighting the difficulty of receiving such information and how situations can change: ‘You’re recontacting but you have no idea what’s going on in that person’s life right now’ (R13). Others highlighted logistical complexities: activation; too many participants; resources. One interviewee, as a solution, wanted all SF so they could initiate recontact himself: ‘If you knew all of the results, that’s more likely to perhaps trigger to you to make contact in years to come if something was discovered’ (R14).

## Discussion

Informed by their experience as patients (or parents) with rare disease, interviewees demonstrated carefully considered and highly pragmatic views towards SF in GS. These interviews illustrated a high level of acceptance *not knowing* secondary findings—this contrasts with previous research demonstrating widespread desire for such results [5].

Participant contentment with not knowing SF appears to stem from extensive experience with the wider healthcare system, which resulted in a high level of confidence in HCPs and trust in the NHS, but also an appreciation of its limitations: interviewees were aware of the costs inherent to healthcare, as well as the complexity of WGS. Focused on finding an answer for their primary condition, many interviewees recalled declining SF. Among those who did wish to receive SF, most were only interested in highly actionable findings. These views—awareness of complexity, focus on actionability—resonate with those expressed by HCPs in the same setting [22]. Although previous studies exploring the views of similar GS participants have shown carefully considered views around SF [13, 14], the levels of circumspection and pragmatism evidenced in the current study appear greater. It is possible that our findings may be a reflection of the experiences of participants actually undergoing GS; a recent survey study—which takes advantage of the contemporaneous enrolment of similar participants—also reported higher rates of SF refusal (>15%) [17], and parents of diagnostic GS recipients in Canada showed ambivalence towards SF (although this was accompanied by a sense of obligation to receive SF for the benefit of their children) [23]. As GS enters routine clinical care, views from a range of participants will be essential to policy development; expectations for SF may differ depending on whether the setting for WGS is research or clinical care. The extent to which the diagnostic odyssey faced by these interviewees informed their views towards SF has implications for informed consent provision: future participants may receive GS as a frontline test, and may thus lack extensive contact with genetics services and the resulting understanding.

In addition, perhaps linked to repeated contact with the healthcare system and explicit long-term nature of the search for primary findings, interviewees expressed raised expectations—namely, that of recontact about SF, and the potential for SF to remain on their medical files should they need to access them later. Coupled with a desire to gain control over their rare disease and the ensuing preferred focus on their primary condition, the assumption that recontact is possible seems to contribute to the above-described comfort not knowing SF. While ethically and clinically desirable, however, recontact in genetics is currently practically unfeasible [24]. UK HCPs perceive significant barriers to the implementation of recontact in clinical genetics in the NHS—namely, limited resources, and a lack of clarity on the division of responsibilities [25]. In response, Dheensa et al. [26] propose a ‘joint venture’ model for recontact, where responsibility is shared between HCPs and participants. The authors encourage debate around the allocation of responsibilities, and urge further research into the technological infrastructure required. In the absence of consistent infrastructure and duty to recontact, our interviewees’ idealistic expectations are over-optimistic: barring (largely unfeasible and currently unplanned) routine genome-wide reanalysis of stored sequence data, the idea that participants could access additional results after declining SF is erroneous, as the majority of these results will never have been generated. This misplaced perception has implications for informed consent provision: in order to make truly informed decisions around SF it is important that those consenting understand the long-term impact of their choices.

Of particular interest is that a majority of interviewees expressed interest in receiving information that could inform reproductive decision-making, including individuals who declined SF. While offered by the 100,000 Genomes Project, screening for carrier status is inconsistent with our own protocol as well as current guidelines [7–9], and warrants consideration in the development of future policies.

Importantly, in order for SF of any kind to be clinically useful they must positively impact outcomes, which might include increasing reproductive choice [27]. Although some commentators hypothesise that SF will be effective in modifying behaviour [28], comments by some participants in this study suggest that this may not necessarily be the case: lack of symptoms and absence of family history may bias recipients to low perceived susceptibility and inaction. Research exploring the behavioural impact of SF disclosure to GS recipients is urgently needed.

On the basis of our experience consenting individuals to GS, it was striking how many recalled not wanting SF. This prompted retrospective comparison (during data analysis) of SF decisions made at the time of consent. In contrast to a survey study by Fernandez et al. [29], which reported no difference in views towards return of SF over an 18-month period, half of our interviewees who felt they would decline SF had originally consented to receive them. This raises questions as to whether their views have since changed or whether the decision at consent was representative of their wishes. It is possible that views have simply changed—time has passed and they may be at a different life stage, which can modulate the impact of such results [30]. It is also possible that they may have reached a greater appreciation of limitations through repeated discussion, including the interview itself—views towards SF have been shown to change during participation in qualitative research [5]. However, it may be relevant that all of these individuals had received positive primary findings at the time of interview. We speculate that some may have felt that declining any analysis, even secondary, could reduce their chances of a diagnosis. Having received a primary finding, desire for SF may have decreased, or participants were able to communicate a lack of desire without fear of comprising a primary diagnosis. Our data neither confirm nor refute this; there is a clear need for research exploring views towards SF over time, particularly before and after receipt of primary results. This has implications for approaches to consent: the distinct nature of analysis for SF from primary findings needs be made clear, and it may also be appropriate to consider introducing mechanisms to better capture and respond to the changing views of participants over time.

### Strengths and limitations

Interviewees are self-selected: those who returned questionnaires and agreed to interviews value research, and tended to have higher levels of education, likely contributing to the high level of understanding. In addition, all interviewees were White British. Despite this, the study captured individuals with a diverse range of primary conditions as well as views towards SF. Furthermore, operating in a very different context than similar US studies [13–16], the sample adds much-needed diversity to a homogeneous literature on views around SF in GS [5].

## Conclusions

Patients with rare disease and parents enroled in GS demonstrated a high level of comfort not knowing SF. This seems to be rooted in a trust in the NHS, informed by experience with rare disease and awareness of the limitations of genetic findings as well as a desire to focus on their primary condition. This was additionally influenced by an expectation for recontact. In the absence of consistent infrastructure for recontact, we argue that responsibilities must be clarified and greater care should be taken at consent to temper expectations. Views around SF appear to be fluid, warranting further research, as well as consideration of innovative approaches to capturing and responding to changing views of patients towards SF over time.

## Electronic supplementary material

Interview guide
