# Supplementary material for: Views of rare disease participants in a UK whole-genome sequencing study towards secondary findings: a qualitative study
Source: Eur J Hum Genet. 2018 Feb 13;26(5):652–9. doi: 10.1038/s41431-018-0106-6 (PMC5945590; doi:10.1038/s41431-018-0106-6)
Supplement: Supplementary file 1 — Interview guide [file 41431_2018_106_MOESM1_ESM.docx]

**MGAC Participant Interview Guide**

**General information**

Please describe the health condition in your family and how it has affected you.

What do you understand about the tests that will be done in this study (called whole-genome or whole-exome sequencing); do you see it a being different from what has been done before? If so in what ways?

What are the differences between whether testing is done as part of clinical care or as part of a research programme? Do you think these differences are important?

What do you hope will come out of this work – for you personally, for your family and more generally?

What are the risks or problems with this kind of test (WGS)? If none, please expand.

**SF: in feedback category**

Were you aware that it was possible for things to be found, in the course of this study, which don’t relate to the condition in the family?

Is that something you’ve thought about? Did you agree on the consent form that you would like to be informed about SF? Do you feel you know enough, then and now, to be able to decide that?

What are the differences between genetic conditions which might affect whether you would want to know about SFs? For example, how serious the condition is, what kind of treatments there are, how likely you would be to develop problems from it

In terms of actionability, we have said we will return findings for which there is some form of treatment available, such as medication or increased screening. What do you think about that?

**SF: not in feedback category**

*Explain: Secondary findings are results that are related to conditions different from the one for which your testing was conducted. We’ve said that we will offer to tell people if we find a genetic variant that suggests likely risk of a serious condition, for which there is some action you could take. Note: do not immediately explain actionability, allowing participant to define this.*

We’ve said we won’t tell you if we find something that is not actionable at present (e.g. Alzheimer’s disease). What do you think about this?

We’ve said we won’t tell you if we find something that we are not sure will mean you are at risk of the condition. What do you think about this?

We’ve said we won’t tell you if we find you are a carrier of a genetic change that would not be important for your health but would be relevant if the person you have children with is also a carrier. What do you think about this?

**Screening for secondary findings**

Today, some studies actively look for things that we think are clinically actionable (such as cancer, heart conditions). What do you think about that?

Who do you think should be involved in the decision about whether to look, and what kind of finding to report back? Why?

Often a panel of experts is involved in making these decisions? How do you feel about that?

**Hypothetical scenario**

*Explain to the interviewee that it may be difficult to imagine, but you would like them to think about a hypothetical scenario*

Is it possible to imagine how you might react if you were told about an SF in your sample? *(Cite examples)*

What would you do with that information? *(Cite examples, probing different types of action)*

In that event, who would you prefer to hear that from?

Who would you tell? Relatives? Who, how?

**Data sharing**

An important part of this research is making your de-identified information available to other researchers in the UK and abroad. What are your concerns about this?

Is there anything else you would like to add?
